# The lethal response to Cdk1 inhibition depends on sister chromatid alignment errors generated by KIF4 and isoform 1 of PRC1

**DOI:** 10.1038/srep14798

**Published:** 2015-10-01

**Authors:** Erik Voets, Judith Marsman, Jeroen Demmers, Roderick Beijersbergen, Rob Wolthuis

**Affiliations:** 1 Division of Cell Biology I (B5) and Division of Molecular Carcinogenesis (B7), The Netherlands Cancer Insitute (NKI-AvL), Plesmanlaan 121, 1066 CX Amsterdam, The Netherlands; 2 Proteomics Center, Erasmus University Medical Center, Dr Molewaterplein 50, 3015 GE Rotterdam, The Netherlands; 3 Section of Oncogenetics, Department of Clinical Genetics and CCA/V-ICI Research Program Oncogenesis, VUmc Medical Faculty, van der Boechorststraat 7, 1081 BT Amsterdam, The Netherlands

## Abstract

Cyclin-dependent kinase 1 (Cdk1) is absolutely essential for cell division. Complete ablation of Cdk1 precludes the entry of G2 phase cells into mitosis, and is early embryonic lethal in mice. Dampening Cdk1 activation, by reducing gene expression or upon treatment with cell-permeable Cdk1 inhibitors, is also detrimental for proliferating cells, but has been associated with defects in mitotic progression, and the formation of aneuploid daughter cells. Here, we used a large-scale RNAi screen to identify the human genes that critically determine the cellular toxicity of Cdk1 inhibition. We show that Cdk1 inhibition leads to fatal sister chromatid alignment errors and mitotic arrest in the spindle checkpoint. These problems start early in mitosis and are alleviated by depletion of isoform 1 of PRC1 (PRC1-1), by gene ablation of its binding partner KIF4, or by abrogation of KIF4 motor activity. Our results show that, normally, Cdk1 activity must rise above the level required for mitotic entry. This prevents KIF4-dependent PRC1-1 translocation to astral microtubule tips and safeguards proper chromosome congression. We conclude that cell death in response to Cdk1 inhibitors directly relates to chromosome alignment defects generated by insufficient repression of PRC1-1 and KIF4 during prometaphase.

At the end of G2 phase, cells enter mitosis when the mitotic kinase cyclin B-Cdk1 is rapidly activated. Simultaneously, the Cdk1-counteracting phosphatase PP2A is inhibited downstream of cyclin B-Cdk1, enforcing phosphorylation of Cdk1 substrates. PP2A can be repressed by a kinase called Greatwall (Gwl)^1^,^2^,^3^,^4^, which is also a Cdk1 substrate^5^,^6^. In mitosis, the chromosomes are bi-oriented on the mitotic spindle. This leads to activation of APC/C^Cdc20^, the ubiquitin ligase that targets cyclin B1 for proteasomal destruction^7^. Disappearance of cyclin B1 inactivates Cdk1. This is essential for mitotic exit and cytokinesis.

Previous work revealed that the majority of cyclin B-Cdk1 is activated before the G2-to-M transition^8^,^9^. Nevertheless, Cdk1 activation continues after nuclear envelope breakdown (NEB), until all the chromosomes are aligned in metaphase^9^,^10^. Cyclin B1 RNAi does not block mitotic entry but interferes with normal prometaphase^9^,^11^,^12^. Also cells that are unable to repress PP2A can enter mitosis even when phosphorylation of Cdk1 substrates remains incomplete^1^,^2^. Similar effects are reported of the highly specific Cdk1 inhibitors purvalanol A and RO-3306: blockade of Cdk1 activity with high concentrations of RO-3306 arrests cells in G2 phase, similar to CDK1 gene ablation^13^,^14^,^15^,^16^. However, lower Cdk1 inhibitor concentrations still permit entry into mitosis but, subsequently, lead to errors during mitotic exit^9^,^11^,^17^. Here, we aimed to identify the molecular pathways that critically determine cellular survival after Cdk1 inhibition, by performing a genome-wide resistance screen for the specific Cdk1 inhibitor RO-3306.

## Results and Discussion

### Optimal cyclin B-Cdk1 activity directs chromosome attachment to the mitotic spindle

**Figure 1:**
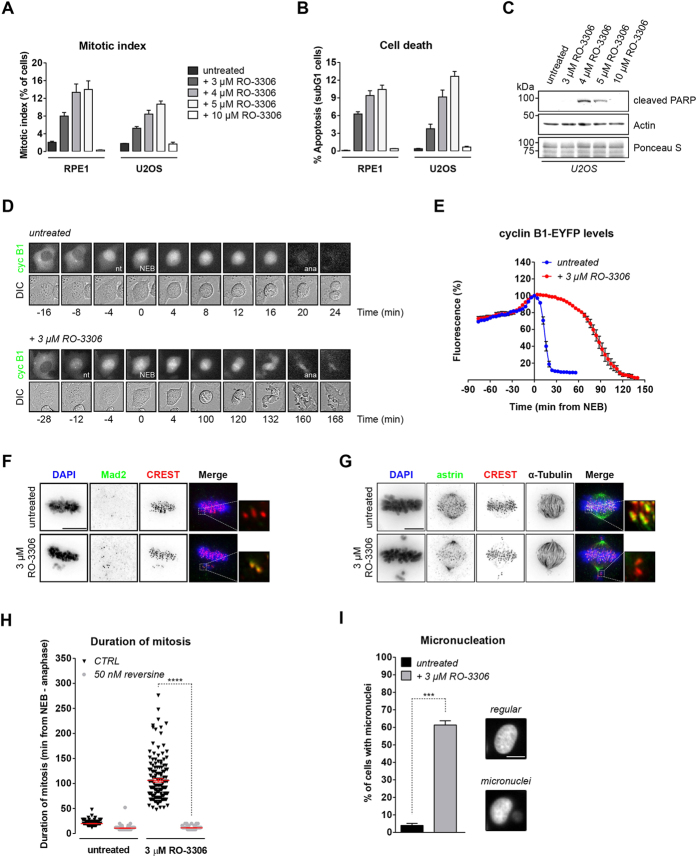
Partial inhibition of Cdk1 results in a spindle checkpoint-dependent mitotic arrest. (**A**) Flow cytometry of RPE1 and U2OS cells treated with increasing concentrations of RO-3306. Cells were treated for 16 hours (Mean ± s.e.m, *n* = 3). (**B**) Cell death correlates to entering mitosis, whereas a full blockade of Cdk1 activity prevents cell death. Cell death was determined by flow cytometry analysis. (Mean ± s.e.m, *n* = 3). (**C**) Partial Cdk1 inhibition triggers PARP cleavage. Lysates of U2OS cells are shown. (**D**) Cyclin B1 degradation in metaphase is delayed in cells with impaired Cdk1 activity. U2OS-CCNB1-EYFP cells were followed by differential interference contrast (DIC) and fluorescence microscopy. NEB, nuclear envelope breakdown; nt, nuclear translocation; ana, anaphase. Scale bar, 10 μm. (**E**) Quantification of the cyclin B1-EYFP levels in cells treated with or without 3 μM RO-3306. Fluorescence intensity was plotted against time after NEB. (Mean ± s.e.m, *n* = 3, untreated = 20 cells analysed, 3 μM RO-3306 treated = 13 cells analysed). (**F**) Unaligned sister chromatids are detected by the mitotic checkpoint. U2OS cells, treated with or without 3 μM RO-3306 were fixed after 16 hours. Mad2-positive kinetochores are indicative of a functional spindle checkpoint. Insets: overlay of Mad2 and CREST stainings. Scale bar, 10 μm. (**G**) Partial Cdk1 inhibition results in sister chromatid attachment defects to the mitotic spindle. Treatments are performed as in (**F**). Sister chromatid pairs that are not aligned are negative for kinetochore-associated astrin. Insets: overlay of astrin and CREST stainings. Scale bar, 10 μm. (**H**) Quantification of the mitotic duration after partial Cdk1 inhibition in presence or absence of a functional checkpoint. Mitosis is plotted from NEB till anaphase. (Mean ± s.e.m, *n* = 3, 50 cells/experiment; ****p < 0.0001, Student’s *t* test). (**I**) Cdk1 inhibition results in the formation of micronuclei during mitotic exit. Analysis is performed using the cells from panel (H). (Mean ± s.e.m, *n* = 3, 50 cells/experiment; ***p < 0.001, Student’s *t* test). Western blots in panel (**C**) have been cropped and full-length gels can be viewed in Supplementary Fig. 6.

We compared the cell cycle effects of different concentrations of the specific Cdk1 inhibitor RO-3306 and found that 16 hours of treatment with RO-3306 at 3–5 μM increased the mitotic index significantly, while more completely blocking Cdk1 activity with 10 μM RO-3306 arrested cells in G2 phase. Similar effects were seen in non-transformed retinal pigment epithelial (RPE1) cells and osteosarcoma (U2OS) cells (Fig. 1A). Interestingly, mitotic arrest after 3–5 μM RO-3306 rapidly led to cell death, while G2 arrest after completely blocking Cdk1 activity was much less detrimental within this time window (Fig. 1B). Cleaved poly-ADP-ribose-polymerase (PARP) revealed the rapid induction of apoptosis after partial, but not after more complete, Cdk1 inhibition (Fig. 1C)^18^.

To investigate the fatal mitotic defects resulting from partial Cdk1 inhibition in more detail, we followed dividing U2OS cells, endogenously tagged with EYFP at the cyclin B1 locus, by time-lapse fluorescence microscopy (U2OS-CCNB1-EYFP)^19^. Low micromolar RO-3306 treatment caused only a short delay between nuclear translocation of cyclin B1 in prophase, and the onset of NEB (Fig. 1D; 12 min in 3 μM RO-3306 versus 4 min in untreated cells). Subsequently however, these cells arrested for approximately 100 minutes before initiating cytokinesis, while cyclin B1-EYFP remained stable (Fig. 1D,E). Cell division eventually started before the completion of anaphase (Fig. 1D). The majority of cells treated with Cdk1 inhibitor displayed multiple chromosomes closely to the spindle poles. These chromosomes stained positive for the spindle checkpoint marker Mad2 (Fig. 1F). The chromosomes were indeed not stably attached to spindle microtubules as revealed by the absence of kinetochore-localized astrin, a marker of plus-end microtubules bound to chromosomes (Fig. 1G)^20^,^21^,^22^. When co-treated with reversine, an Mps1 inhibitor that blocks signalling from unattached kinetochores^23^, the arrested cells rapidly exited mitosis without aligning their chromosomes, regardless of Cdk1 inhibition (Fig. 1H, Supplementary Fig. 1A; mitosis lasted 106 ± 3.46 min in 3 μM RO-3306 or 11.3 ± 0.25 min in 3 μM RO-3306 + 50 nM reversine). In the absence of reversine, this led to unequal segregation of sister chromatids and caused aneuploidy, often accompanied by the formation of micronuclei (Fig. 1I; 4 ± 1.16% micronucleated cells in untreated cells versus 61.3 ± 2.4% in 3 μM RO-3306). Most of the RPE1 cells that left mitosis after prolonged Cdk1 inhibition eventually arrested in a 4n G1-like state. Partial depletion of Cdk1 or B-type cyclins by RNAi lead to identical defects in cell cycle progression and nuclear morphology as observed after prolonged RO-3306 treatment, providing important evidence that treatment with the inhibitor for several days specifically represses cyclin B-Cdk1 (Supplementary Fig. 2A–G)^9^,^11^,^12^,^24^. Taken together, these results show that optimal mitotic Cdk1 activity is particularly required to align the sister chromatids, inactivate the spindle checkpoint, and degrade cyclin B1. This may be surprising because cyclin B-Cdk1 reportedly has a positive role in maintenance of the spindle checkpoint, too^11^,^25^. We investigated this paradox by measuring how long cells arrested when entering mitosis in the presence of nocodazole alone, which arrests cells in the spindle checkpoint, or in the combination of nocodazole with 3 μM RO-3306. Partial inhibition of Cdk1 halved the duration of the nocodazole-induced delay. This showed that the spindle checkpoint is functional in cells entering mitosis with suboptimal Cdk1 activity, but maintenance of the checkpoint is weakened (Supplementary Fig. 1B,C; mitosis lasts 473 ± 12.6 min in nocodazole or 240.8 ± 9.6 min in nocodazole + 3 μM RO-3306). Collectively, these data demonstrate that incomplete activation of cyclin B-Cdk1 during mitosis perturbs chromosome alignment and subsequent satisfaction of the spindle checkpoint, and leads either to apoptosis, or, when cells eventually divide, to aneuploidy.

### A role for PRC1 splice variant 1 in RO-3306 resistance, independent of cytokinesis

**Figure 2:**
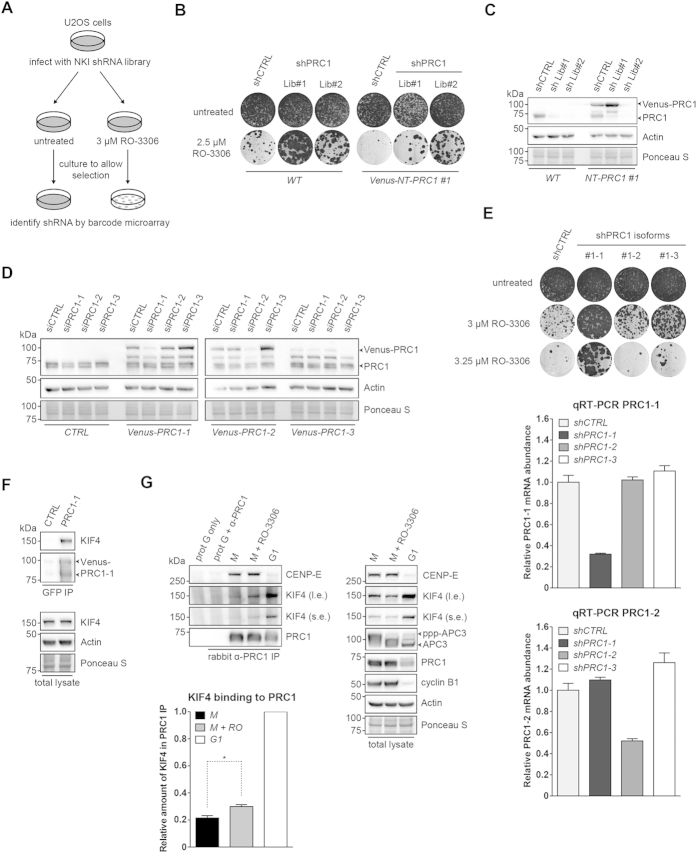
A shRNA barcode screen identifies PRC1 as critical mediator of the anti-proliferative effects of RO-3306. (**A**) Schematic outline of the RO-3306 barcode screen in U2OS cells. (**B**) Reconstitution of the RNAi-resistant human PRC1-1 cDNA in PRC1 knockdown cells. The pRS vector (shCTRL) was used as a control. (**C**) Western blot analysis of protein lysates corresponding to the cell lines shown in (**B**). shPRC1 Lib#1 targets only endogenous PRC1, while shPRC1 Lib#2 is able to target both endogenous and Venus-NT-PRC1 #1. (**D**) Western blot analysis of U2OS cells stably expressing individual Venus-PRC1 isoforms after transfection of siRNA duplexes specifically targeting the indicated splice variants. CTRL refers to cells lacking ectopic expression of Venus-PRC1. (**E**) Depletion of PRC1-1 rescues cell proliferation when Cdk1 activity is compromised. The functional phenotypes of the individual shRNAs targeting the PRC1 variants are indicated by the colony formation assay. The pRS vector (shCTRL) was used as a control. The knockdown efficiency of each individual shRNA was measured by examining the mRNA level of the PRC1-1 and PRC1-2 target genes by qRT-PCR. (Mean ± s.d. *n* = 3). (**F**) KIF4 is a PRC1 binding partner. Immunoprecipitations (IPs) with GFP-Trap_A beads were performed on extracts of U2OS WT (CTRL) or U2OS cells stably expressing Venus-PRC1-1 (PRC1-1). Aliquots of the IPs were analysed by SDS-PAGE. (**G**) Compromised Cdk1 activity in mitosis results in premature binding of PRC1 to KIF4. U2OS cells were synchronised in mitosis using thymidine and nocodazole. Cells arrested in mitosis were obtained by mitotic shake-off, released from the nocodazole and harvested after 60 minutes (G1) or, alternatively, treated with 5 μM MG132 with or without 3 μM RO-3306 and harvested after 30 minutes (M). Cell extracts were subjected to immunoprecipitation using a rabbit anti-PRC1. Aliquots of the immunoprecipitates were analysed by Western blotting. The signal intensity of the KIF4 band in the PRC1 IP was quantified and normalized to 1 for the G1 sample. *p < 0.05, Student’s *t* test. Western blots in panels (**C**,**D**,**F**,**G**) have been cropped and full-length gels can be viewed in Supplementary Fig. 6.

To find the genes most critically involved in RO-3306-induced lethality, we then carried out a large-scale RNAi screen with a collection of 23,742 short hairpin RNA (shRNA) vectors targeting 7,914 human genes^26^. Barcoding technology was used to identify genes whose knockdown supported survival and proliferation in the presence of 3 μM RO-3306 (Fig. 2A). The results are shown in an M/A-plot, where each dot represents one individual shRNA vector (Supplementary Fig. 3A). M and A values reflect relative enrichment and hybridization signal intensities. We prioritized our candidates by applying the M/A cut-off values as indicated. Among the candidate hits, we identified short hairpins targeting a scaffold subunit of the PP2Aα complex, PPP2R1A, and protein regulator of cytokinesis 1 (PRC1), as mediators of RO-3306 resistance (Supplementary Fig. 3A). Each of the identified shRNAs were then retested for their ability to confer resistance to RO-3306, in comparison to a positive control, a shRNA targeting PPP2CA (the catalytic subunit of PP2Aα). Of the shRNA vectors tested, the strongest bypass of RO-3306 sensitivity was conferred by shPRC1 or shPPP2CA, and, partially, by shPPP2R1A (Supplementary Fig. 3B,C). Conferred resistance by different knockdown vectors correlated with reduced expression of PRC1 (Supplementary Fig. 3D,E). Expression of a shRNA-resistant PRC1 cDNA in PRC1-depleted U2OS cells restored the sensitivity of these cells to Cdk1 inhibition, further supporting the specificity of the PRC1 shRNAs (Fig. 2B,C).

PRC1 is a well-conserved microtubule-associated protein. In mammalian cells, it plays a vital role in the establishment of the spindle midzone, required for cytokinesis^27^. Mammalian cells express three different PRC1 splice variants. These variants have different carboxyl termini. PRC1-2 is activated by Plk1 phosphorylation and controls cytokinesis^28^. To further address the contribution of the PRC1 splice variants to RO-3306 sensitivity, siRNA duplexes were designed which specifically depleted each of the different PRC1 isoforms (Fig. 2D). Depletion of either PRC1-1 or PRC1-3 allowed cells to bypass Cdk1 inhibition. PRC1-1 depletion conferred the strongest resistance to higher doses of RO-3306 (Fig. 2E). Importantly, on its turn, only PRC1-2 cDNA expression restored the cytokinesis defects that were caused by depletion of all PRC1 isoforms (Supplementary Fig. 4A,B). Furthermore, overexpression of PRC1-2 did not sensitize U2OS cells to Cdk1 inhibition, while overexpression of PRC1-1 clearly did (Supplementary Fig. 4C,D). These results identify PRC1, and particularly the isoform PRC1-1, as a factor that critically determines the cellular sensitivity to Cdk1 inhibition.

### Removal of the PRC1 interactor KIF4 restores mitosis after Cdk1 inhibition

To gain further insight into the role of PRC1-1, we conducted a tandem mass spectrometry experiment to find its interacting proteins. We identified many of the known PRC1 binding partners, but particularly the chromokinesin KIF4^29^ (Supplementary Fig. 5A). Western blotting of anti-GFP immunoprecipitations confirmed the interaction between Venus-PRC1-1 and endogenous KIF4 (Fig. 2F). Partial inhibition of Cdk1 in mitotic cells resulted in KIF4-PRC1 protein complex formation and was further increased after mitosis, when Cdk1 is inactive (Fig. 2G, left panel; right panel shows expression levels).

**Figure 3:**
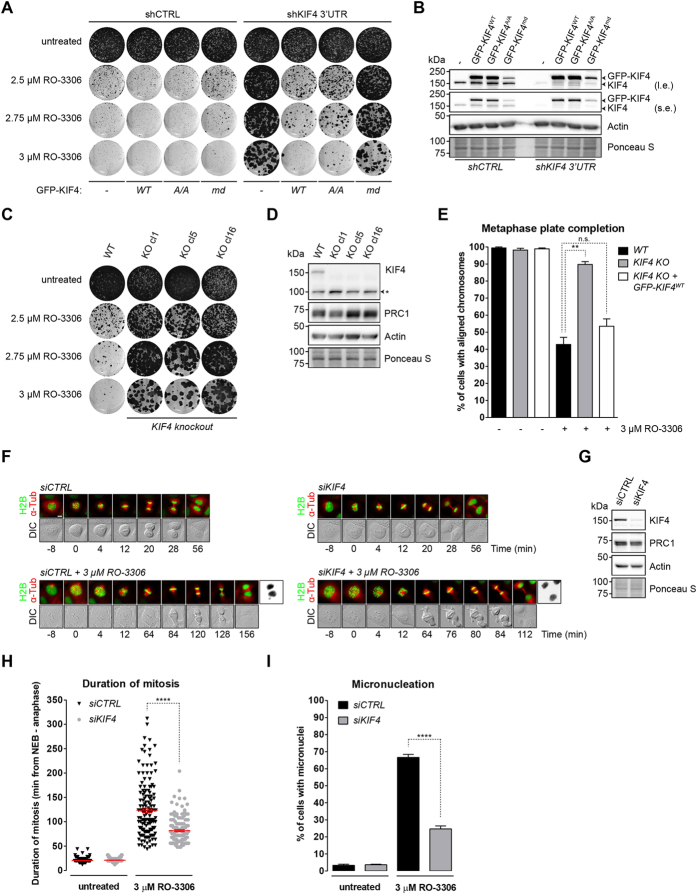
Suppression of the PRC1 interactor KIF4 improves the mitotic cell fate when Cdk1 activity is compromised. (**A**) The ATPase activity of KIF4 is required for the RO-3306–induced cellular toxicity. Reconstitution of KIF4 knockdown U2OS cells with either WT, a phosphorylation-mutant, or an ATPase-dead KIF4 cDNA (KIF4^WT^, KIF4^A/A^, or KIF4^md^, respectively). (**B**) Western blot analysis of protein lysates corresponding to the cell lines shown in (**A**). shKIF4 3′UTR targets endogenous KIF4. (**C**) KIF4 knockout cells are resistant to Cdk1 inhibition. U2OS WT or KIF4 knockout cell clones treated with increasing concentrations of RO-3306 were examined by colony formation assay. KO, knockout. (**D**) U2OS WT and KIF4 KO clones were characterized by Western blot analysis. Cell lysates correspond to the colony formation assay in (**C**). (**E**) KIF4 KO cells restore chromosome alignment in mitosis when Cdk1 activity is compromised. U2OS WT, KIF4 KO clone 16, and KIF4 KO clone 16 reconstituted with GFP-KIF4^WT^ were treated with or without 3 μM RO-3306 for 16 hours and MG132 for two hours to allow cells to arrest in metaphase. Subsequently, cells were fixed and the alignment status was determined by immunofluorescence. (Mean ± s.e.m, *n* = 3, at least 50 cells/experiment; n.s., not significant; **p < 0.005, Student’s *t* test). (**F**) U2OS cells stably expressing H2B-GFP and mCherry-α-Tubulin were imaged by DIC and fluorescence microscopy, 48 hours after transfection with indicated siRNAs. Scale bar, 10 μm. (**G**) Verification of the KIF4 depletion judged by Western blot analysis. (**H**) KIF4 depletion rescues the mitotic delay upon compromised Cdk1 activity. Cells shown in (**F**) were followed 48 hours after transfection with indicated siRNAs. Mitosis is plotted from NEB until anaphase. (Mean ± s.e.m, *n* = 3, 50 cells/experiment; ****p < 0.0001, Student’s *t* test). (**I**) KIF4 knockdown rescues abnormal mitotic exit and chromosome loss when Cdk1 is partially active. Analysis is performed with cells shown in (**H**). (Mean ± s.e.m, *n* = 3, 50 cells/experiment; ****p < 0.0001, Student’s *t* test). Western blots in panels (**B**,**D**,**G**) have been cropped and full-length gels can be viewed in Supplementary Fig. 6.

We then tested the effect of KIF4 knockdown by multiple independent shRNAs, which all rendered cells resistant to RO-3306, similar to the knockdown of PRC1 (Supplementary Fig. 5B,C). Furthermore, reconstitution of KIF4 knockdown U2OS cells by the RNAi-resistant human WT KIF4 cDNA fully restored the sensitivity of these cells to Cdk1 inhibition (Fig. 3A,B). Active KIF4 inhibits microtubule plus-end dynamics to minimize the length of overlapping PRC1-bound microtubules within the spindle midzone^30^,^31^,^32^. To test whether the RO-3306–induced lethality also depended on the motor activity of KIF4, we expressed two different KIF4 mutants to complement KIF4 knockdown cells: a phosphorylation mutant that is expected to be less active in mitosis (KIF4^A/A^)^33^, and an ATPase inactive or motor-dead mutant (KIF4^md^)^34^. This experiment showed that the growth inhibitory effects of RO-3306 strongly depended on the ATPase activity of KIF4 (Fig. 3A,B). The A/A mutant was limited in its ability to restore RO-3306 sensitivity, in line with its moderately impaired motor activity^33^. Reciprocally, overexpression of GFP-tagged human or mouse KIF4 cDNA was sufficient to increase the toxicity of RO-3306 in U2OS cells (Supplementary Fig. 5D,E). So, reducing the expression of KIF4 renders Cdk1 inhibition less toxic, while increasing the abundance of KIF4 aggravates toxicity. To prove the specificity of the KIF4 phenotype, we disrupted the KIF4 gene in U2OS cells using CRISPR/Cas-mediated gene targeting. Successful gene ablation resulted in the disruption of exon 2 of the KIF4 gene by a blasticidin resistance (Blast^R^) gene (Supplementary Fig. 5F). In addition, immunostaining revealed the complete loss of the KIF4 protein (Supplementary Fig. 5G). Despite some clonal differences, gene ablation of KIF4 again rescued cellular proliferation in the presence of low doses of RO-3306 (Fig. 3C,D). Further characterization of one of these KIF4 knockout clones (KIF4 KO clone 16) demonstrated that loss of KIF4 rescued chromosome alignment under conditions when Cdk1 was partially inhibited (Fig. 3E, Supplementary Fig. 5H).

To study the spindle, and chromosome alignment, in further detail, we depleted KIF4 from a stable cell line co-expressing the markers H2B-GFP and mCherry-α-Tubulin. KIF4 depletion resulted in an elongated spindle midzone when compared to control cells, an effect published before^35^ (Fig. 3F). Western blotting confirmed that KIF4 was not expressed (Fig. 3G). Importantly, KIF4 RNAi shortened the duration of prometaphase in RO-3306 (Fig. 3H; siCTRL, 123.7 ± 4.65 min; si*KIF4*, 81.7 ± 2.16 min). At the same time, KIF4 depletion reduced the formation of micronucleated cells significantly (Fig. 3I; siCTRL, 66.7 ± 1.76%; siKIF4, 24.7 ± 1.76%). Under untreated conditions, we did not observe any change in the duration of NEB until anaphase, or the amount of micronucleated daughter cell formation when KIF4 was suppressed. These data indicate that PRC1 and KIF4 act in a similar fashion and critically mediate the inhibitory effects of RO-3306 on mitosis. We conclude that, like the suppression of PRC1-1, removal of KIF4 restores chromosome alignment, cell viability and genomic integrity when Cdk1 is inhibited.

### KIF4 directs chromosome alignment by regulating the stability of the mitotic spindle

**Figure 4:**
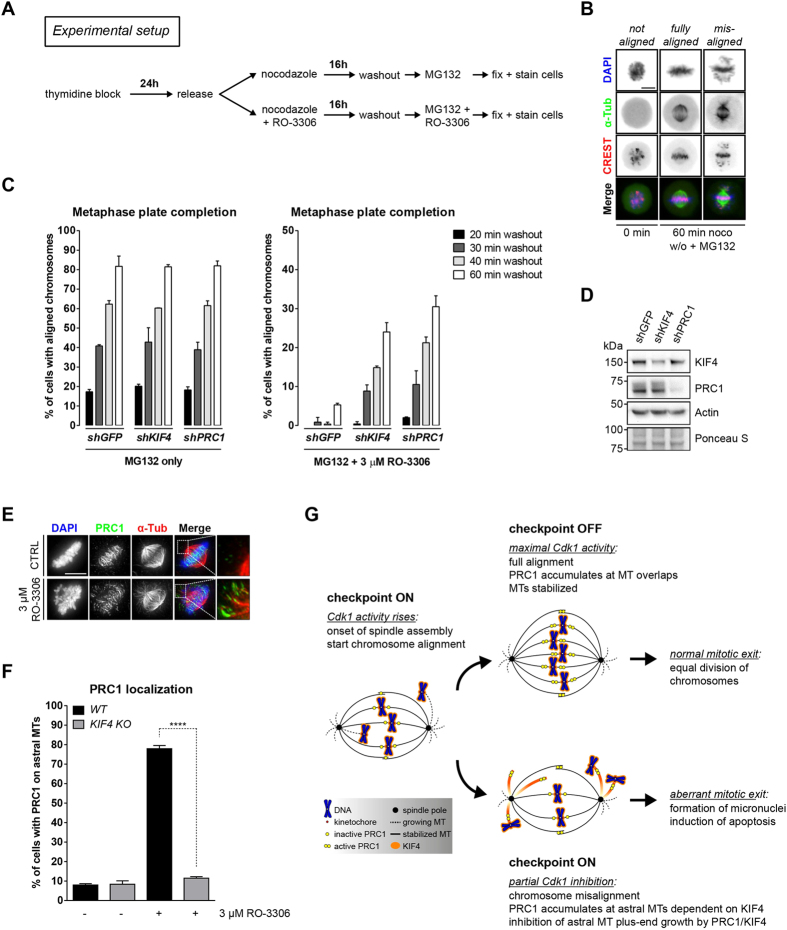
KIF4 and PRC1 repress chromosome congression when Cdk1 is partially active. (**A**) Experimental setup of a nocodazole washout assay. Thymidine blocked cells are released, nocodazole-arrested, and washed free from nocodazole to allow spindle assembly in presence of the proteasome inhibitor MG132, together with RO-3306. (**B**) Representative immunofluorescence images of mitotic cells, before and after nocodazole washout. Examples of different alignment defects are shown. Scale bar, 10 μm. (**C**) PRC1 and KIF4 prevent chromosome alignment when Cdk1 activity is compromised. Bar graph shows a quantification of the experimental setup shown in (**A**). (Mean ± s.e.m, *n* = 3, at least 150 cells/experiment). (**D**) Western blot analysis of the experiment in (**C**) shows the knockdown efficiency of the shRNAs targeting KIF4 or PRC1. (**E**) PRC1 present at microtubule overlaps partly localizes to astral microtubules after suppression of Cdk1 activity. U2OS cells were treated with or without 3 μM RO-3306 for 16 hours, fixed, and processed for immunofluorescence. Insets demonstrate PRC1 localization to astral microtubules in the vicinity of unaligned chromosomes when Cdk1 is inhibited. Scale bar, 10 μm. (**F**) PRC1 localization to astral microtubules is dependent on the presence of KIF4. U2OS WT or KIF4 KO cells were treated and processed as in (**E**). Prometaphase cells were scored for the presence of PRC1 on astral microtubules (MTs). Scale bar, 10 μm. (Mean ± s.e.m, *n* = 3, at least 30 cells/experiment; ****p < 0.0001, Student’s *t* test). (**G**) Schematic overview of the progression from prometaphase to metaphase with normal or compromised cyclin B-Cdk1 activity. Maximal Cdk1 activation directs chromosome alignment and the establishment of stable k-MT attachments in metaphase. Partial reduction in Cdk1 activity by RO-3306 treatment results in a prometaphase arrest that is dependent on KIF4 and PRC1. Due to a weakened spindle checkpoint, cells will exit from mitosis with lagging chromosomes leading to the formation of micronuclei. Western blots in panel (**D**) have been cropped and full-length gels can be viewed in Supplementary Fig. 6.

Next, we investigated whether depletion of KIF4 or PRC1 affected the dynamics of spindle formation and chromosome alignment in RO-3306. We conducted a nocodazole washout assay that reveals how efficiently kinetochores can establish stable microtubule attachments as the spindle forms (Fig. 4A). At time points up to 60 min after nocodazole washout, we measured the fraction of cells with fully aligned chromosomes at the metaphase plate. Examples of misaligned and fully aligned chromosomes are shown in Fig. 4B. The dynamics of alignment were unchanged in the absence of RO-3306 when either KIF4 or PRC1 was suppressed (Fig. 4C, left panel). In control cells, partial inhibition of Cdk1 completely abolished alignment of the chromosomes up to 40 min after nocodazole washout, but depletion of either KIF4 or PRC1 clearly restored alignment under these conditions (Fig. 4C, right panel). Western blot analysis confirmed the knockdown of both KIF4 and PRC1 (Fig. 4D). When cells entered mitosis under conditions of partial Cdk1 inhibition, PRC1 remained incompletely phosphorylated on Thr^481^ during prometaphase and was recruited to misaligned chromosomes as well as what appear to be astral microtubule tips: microtubules moving away from the mitotic spindle towards the cell cortex (Fig. 4E,F, Supplementary Fig. 4E,F). The effect of RO-3306 on PRC1 was completely dependent on KIF4 (Fig. 4F). We conclude that, normally, PRC1 and KIF4 are kept inactive in early mitosis by Cdk1, which is essential for the prometaphase-to-metaphase transition and for successful mitosis^33^,^36^. Entering mitosis in the presence of Cdk1 inhibitors renders KIF4 abnormally active, directing the translocation of PRC1-1 to astral microtubule tips. This interferes with the formation of stable k-MT attachments, leading to chromosome alignment defects and a spindle checkpoint-dependent arrest, and results in cell death or aneuploidy (Fig. 4G).

## Conclusions

Our results reveal that Cdk1 inhibition triggers cell death, in line with previous reports^12^,^14^,^16^,^37^,^38^,^39^. Importantly, we demonstrate that short-term treatment with a low dose of RO-3306 induces mitotic arrest and cell death, or aneuploidy, and this occurs independently of endoreduplication cycles. Mitotic failure may lead to nuclear fragmentation, thereby generating daughter cells with characteristic micronuclei. These micronuclei are a well-known hallmark of chromosomal instability (CIN) and aneuploidy^40^,^41^. Micronucleated cells are often eliminated by caspase-3-mediated apoptosis^42^. PRC1-1 or KIF4 depletion reduces the amount of micronucleated cells, which may explain the increased cell viability upon RO-3306 treatment. Additionally, PRC1-1 and KIF4 RNAi facilitate sister chromatid alignment and passage through the spindle checkpoint, vitally shortening the duration of mitosis in the presence of RO-3306 and preventing the accumulation of death signals in mitosis^43^.

Thus far, it was unclear which Cdk1-regulated factors direct the timing of k-MT attachment stabilization. Our results reveal that PRC1 and KIF4 each fit this role. PRC1 is a Cdk1 substrate that is repressed by phosphorylation of both Thr^470^ and Thr^481^ during prometaphase, preventing its oligomerization and microtubule bundling activity^27^,^36^,^44^. The inability to phosphorylate PRC1 causes defects in chromosome alignment and an arrest in mitosis^36^. Remarkably, our results support a cytokinesis-independent role of PRC1 in cell survival after Cdk1 inhibition: we find that PRC1-1 is the key PRC1 splice variant that contributes to RO-3306 sensitivity from prometaphase onwards. The localization of PRC1-1 drastically changes upon partial inhibition of Cdk1, from microtubules at overlapping spindle halves, to both polar and astral microtubules.

KIF4 has a binding preference for dephosphorylated PRC1, so the interaction between KIF4 and PRC1 normally occurs after cyclin B destruction and PP2A-B55 activation^34^,^45^. Thus far, we have no evidence for direct control of KIF4 by Cdk1. We hypothesize that, under conditions of Cdk1 inhibition, KIF4 reduces the growth of anti-parallel microtubule overlaps, formed by PRC1 in prometaphase. This suppresses the dynamic growth of microtubule plus-ends, leading to an inability to capture chromosomes and congress them to the metaphase plate.

In initial clinical trials, maximal inhibition of Cdk1 did not appear to be very successful in the treatment of human cancers. However, Cdk1 inhibition is relatively efficient in the treatment of Myc-driven tumors^46^. Possibly, Cdk1 inhibition works better as an anti-cancer strategy when the targeted cells do not arrest in G2 phase, but undergo a fatal mitosis. The expression levels of PRC1-1 and KIF4 in tumors could be factors influencing the outcome of treatment with Cdk1 inhibitors. These ideas will be the subject of further studies.

## Methods

### Cell culture and synchronization

Cells lines were cultured in DMEM (Gibco) containing 8% heat-inactivated FBS (Hyclone) supplemented with 100 U/ml penicillin and 100 μg/ml streptomycin at 37 °C in 5% CO_2_. 24 or 48 hours before synchronization, transfection or treatment, cells were seeded on either 6-well Costar plates or 9 cm Falcon dishes. For time-lapse fluorescence microscopy, cells were seeded onto 3.5 mm glass-bottom dishes (Wilco Wells) or 4- to 8-well glass-bottom dishes (Labtek II). For enrichment of cells in mitosis, cells were treated for 24 hours with thymidine (2.5 mM final concentration [Sigma-Aldrich]), released into fresh medium and subsequently treated with nocodazole (415 nM final concentration [Sigma-Aldrich]) for 16 hours. For alignment assays, cells were released from the nocodazole block and cultured in medium containing MG132 (#13697, 5 μM final concentration [Cayman Chemicals]) to block the cells in metaphase.

Other drugs in this study are used as indicated: RO-3306 (#217699 [Calbiochem]); Mps1 inhibitor reversine (#10004412, 50 nM final concentration [Cayman Chemicals]).

### shRNA library screen

U2OS cells were infected with retroviruses representing the complete Netherlands Cancer Institute (NKI) shRNA library described previously^26^. In brief, infected cells were selected on puromycin (2.0 μg/ml) for three days and split into two populations. One population was left untreated and harvested after 7 days, while the other population was continuously exposed to the Cdk1 inhibitor RO-3306 and harvested after 22 days. The barcode screen was performed as described previously^26^,^47^.

### Plasmids and siRNA

Homo sapiens cDNAs for KIF4 and the different PRC1 splice variants were PCR amplified from pcDNA5/FRT/TO/EGFP-KIF4 and pCRII.topo-PRC1, respectively and cloned into pEGFP-C1 or pVenus-C1 (Takara Bio Inc.) using BglII-HindIII or EcoRI-SalI sites. The viral plasmids pLIB-GFP-KIF4 and pLIB-Venus-PRC1-1/-2/-3 were constructed by subcloning the inserts into a modified pLIB vector. Mutants of PRC1 and KIF4 were made by site-directed mutagenesis using the Venus-PRC1 isoforms or GFP-KIF4 as a template. The GFP-tagged versions of KIF4^A/A^ and KIF4^md^ were generated as described^33^,^34^. A *Mus musculus* variant of KIF4 was subcloned from pEGFP-mKIF4 to generate pLIB-GFP-mKIF4.

For the generation of pRetroSuper-Puro vectors targeting human KIF4, PPP2CA, PPP2R1A, and PRC1, the 19- or 21-nucleotide sequences were used as described in Supplementary Table 1. The PRC1 isoforms were targeted with 19-nucleotide sequences designed to match the unique splice junctions as described previously^28^. The shPRC1 pool refers to a combination of 4 individual shRNAs (e.g. shPRC1#2, #5, Lib#1, and Lib#2) targeting PRC1.

The siRNAs to target KIF4 or PRC1 were purchased from Thermo Fisher Scientific as set of four individual ON-TARGET-plus oligos (see Supplementary Table 2).

### Transfections and infections

U2OS cells stably expressing H2B-GFP and mCherry-α-Tubulin were generated by retroviral infection with pRS-H2B-GFP and pCX-mCherry-α-Tubulin, respectively. Cell lines expressing GFP-KIF4 or the different Venus-PRC1 splice variants were generated by retroviral infection with either modified versions of pLIB-GFP-KIF4 or the pLIB-Venus-PRC1 splice variants.

A CRISPR sgRNA was designed (using the CRISPR tool available at http://crispr.mit.edu/) targeting the human KIF4 gene (exon 2; 5′-ATTTGATCCCTCTACTGAAC-3′) and cloned into the BbsI site of PX330 (http://www.addgene.org/42230/)^48^. U2OS cells were transfected with the gene-specific PX330 vector in addition to an excision vector containing a sgRNA to the zebrafish TIA gene (5′-GGTATGTCGGGAACCTCTCC-3′) and a cassette of a 2A sequence followed by a blasticidin resistance gene, flanked by two TIA target sites. Co-transfection with PX330 results in excision of the cassette from the plasmid and subsequent sporadic incorporation at the site of the targeted genomic locus by non-homologous end joining^49^. Transfections were carried out using X-tremeGENE 9 (Roche) according to the manufacturer’s instructions. Successful integration of the cassette into the targeted gene disrupts the allele and renders cells resistant to blasticidin. Four days following transfection the culture medium was supplemented with blasticidin (10 mg/ml). Surviving colonies were clonally expanded and screened for cassette integration into the query gene by PCR.

For siRNA transfections, cells were seeded into 6-well plates and transfected twice (with a 24 hour time-interval) using 40 nM siRNA. Transfection of siRNA pools were performed using Lipofectamine 2000 (Invitrogen) according to the manufacturer’s instructions. For time-lapse microscopy, cells were seeded into Labtek II 4- to 8-well glass-bottom dishes after the second siRNA transfection, or alternatively, harvested for Western blot analysis.

### Antibodies

Antibodies against the following proteins were used: goat anti-actin (Santa Cruz sc-1616), mouse anti-α-Tubulin (Sigma T5168), ANA-Centromere CREST AutoAb Human (Fitzgerald 90C-CS1058), mouse anti-APC3 (BD Transduction #610455), rabbit anti-astrin (Bethyl Laboratories A301-512A), mouse anti-Cdk1 (BD Transduction 610038), rabbit anti-Cdk2 (Santa Cruz sc-163), rabbit anti-CENP-E (Sigma C7488), rabbit anti-cleaved PARP (Cell Signaling #5625S), mouse anti-cyclin B1 (Santa Cruz sc-245), rabbit anti-cyclin B2 (Santa Cruz sc-22776), rabbit anti-KIF4 (Bethyl Laboratories A301-074A), rabbit anti-Mad2 (Bethyl Laboratories A300-301A), goat anti-phospho-PRC1 Thr^481^ (Santa Cruz sc-11768), mouse anti-PRC1 (BioLegend 629002), rabbit anti-PRC1 (Santa Cruz sc-8356). Secondary peroxidase-conjugated antibodies were obtained from DAKO and ALEXA fluorescently-labelled secondary antibodies were purchased from Molecular Probes.

### Flow cytometry

For analysis of the cell cycle distribution, the entire cell population was analyzed by harvesting the medium containing the fraction of floating cells (e.g. dead and mitotic cells) together with the adherent cells (using 0.05% Trypsin-EDTA to detach the cells). Subsequently, cells were washed two times with PBS containing 0.01% Tween-20. Finally, the cells were fixed in 70% ice-cold ethanol and stained with rabbit anti-phospho-Histone H3 Ser^10^ (#06-570, 1:400 [EMD Millipore]). Cells were counterstained with propidium iodide (Sigma) diluted in RNase-containing PBS. Phospho-Histone H3 positivity of the cells was analysed using FCS Express 2 (De Novo Software).

### Western blotting and immunoprecipitations

Cells were lysed in ELB + (150 mM NaCl, 50 mM HEPES (pH 7.5), 5 mM EDTA, 0.3% NP-40, 10 mM β-glycerophosphate, 6% glycerol, 5 mM NaF, 1 mM Na_3_VO_4_ and Roche protease inhibitor cocktail). Lysates were cleared by centrifugation (13,000 × g, 10 min at 4 °C). Protein levels were equalized by Bradford assay. For immunoprecipitations, 2 μg antibodies were precoupled for 4–12 hours to 20 μl of protein G Sepharose (Amersham Biosciences) and washed with ELB + . Precoupled beads and lysates were incubated for 4 hours at 4 °C and washed three times with 1.0 mL of ice-cold ELB + . All remaining buffer was then removed and beads were resuspended in 50 μL Laemmli sample buffer; 25 μL was separated on SDS-PAGE and blotted on nitrocellulose (0.4 μm pore). Immunoprecipitations of GFP for mass spectrometry were performed with GFP-Trap_A beads (Chromotek), according to the manufacturer’s protocol. Membranes were blocked with 5% ELK in PBS containing 0.1% Tween. Development of blots was performed using the Chemidoc Imaging System (Bio-Rad Laboratories) and quantification was done with the Image Lab (Bio-Rad Laboratories) software.

### Immunofluorescence

Cells were grown on glass coverslips and fixed with 3.7% formaldehyde in PBS (containing Ca^2+^ and Mg^2+^) for 10 min followed by permeabilization for 10 min with 0.1% Triton X-100 in PBS (Ca^2+^ and Mg^2+^). Cells were blocked in PBS (Ca^2+^ and Mg^2+^) containing 10% FBS and labelled with primary antibodies as indicated. Cells were washed 3 times followed by labelling with Alexa Fluor secondary antibodies. Staining of F-actin was carried out using Phalloidin-568 (Molecular Probes). DNA staining was performed with 4′,6-Diamidino-2-phenylindole (DAPI [Molecular Probes]) after which the coverslips were mounted in Vectashield solution. For endogenous KIF4 and PRC1 stainings, cells were pre-permeabilized for 3–5 min in PHEM buffer (100 mM PIPES pH 6.8, 25 mM HEPES pH 7.4, 5 mM EGTA, 2.5 mM MgCl_2_) plus 0.5% Triton X-100. Then, cells were fixed for 10 min in PHEM buffer containing 3.7% formaldehyde. Mitotic PRC1 phosphorylation was monitored after ice-cold methanol fixation. Calcium-stable microtubule stainings were carried out as described previously^50^. Subsequent steps were performed as described above. z-stacks with 0.2 μm spacing were acquired using a DeltaVision Elite system (Applied Precision). Maximum intensity projection of the z-levels were analysed with ImageJ (National Institute of Health) and processed using Photoshop and Illustrator software (Adobe).

### Time-lapse fluorescence microscopy

U2OS cells were transfected with indicated siRNAs and followed by fluorescence time-lapse microscopy. Acquisition of DIC and fluorescence images started 24 or 48 hours after transfection on an automated microscope (Axio Observer Z1; Carl Zeiss) in a heated culture chamber (5% CO_2_ at 37 °C) using DMEM with 8% FCS and antibiotics. The microscope was equipped with an LD 0.55 condenser and 40x NA 1.40 Plan Apochromat oil DIC objective and CFP/YFP and GFP/HcRed filter blocks (Carl Zeiss) to select specific fluorescence. Images were taken using AxioVision Rel. 4.8.1 software (Carl Zeiss) with a charge-coupled device camera (ORCA R2 Black and White CCD [Hamamatsu Photonics] or Roper HQ [Roper Scientific]) at 100-ms exposure times. Alternatively, imaging was performed on a Deltavision Elite system, using Leibovitz’s L-15 (Gibco) CO_2_-independent medium, in a 37 °C culture chamber. For quantitative analysis of degradation, MetaMorph software (Universal Imaging), ImageJ and Excel (Microsoft) were used. Captured images were processed using Photoshop and Illustrator software.

### RNA isolation and qRT-PCR analysis

Total RNA was extracted from U2OS cells grown on 6-well plates using the Quick-RNA MiniPrep kit (Zymo research) and quantified using NanoDrop (Thermo Fisher Scientific). cDNA was synthesized using SuperScript II reverse transcription, random hexamer primers (Promega), and 1 μg of total RNA according to the manufacturer’s protocol. Primers were designed with a melting temperature close to 60 °C to generate 90–120 bp amplicons, mostly spanning introns. cDNA was amplified for 40 cycles on a 7500 Fast Real-Time PCR System (Applied Biosystems) using PCR mix reaction (FastStart Universal SYBR green mastermix [Roche]). Target cDNA levels were analysed by the comparative cycle (Ct) method and values were normalized against glyceraldehyde-3-phosphate dehydrogenase (GAPDH) expression levels. Information regarding the nucleotide sequences used for qRT-PCR analysis can be found in Supplementary Table 3.

## Additional Information

**How to cite this article**: Voets, E. *et al*. The lethal response to Cdk1 inhibition depends on sister chromatid alignment errors generated by KIF4 and isoform 1 of PRC1. *Sci. Rep*. **5**, 14798; doi: 10.1038/srep14798 (2015).

## Supplementary Material

Supplementary Information

## References

[b1] Voets E. & Wolthuis R. M. F. MASTL is the human orthologue of greatwall kinase that facilitates mitotic entry, anaphase and cytokinesis. Cell Cycle 9, 3591–3601 (2010). 20818157 10.4161/cc.9.17.12832

[b2] Burgess A. . Loss of human Greatwall results in G2 arrest and multiple mitotic defects due to deregulation of the cyclin B-Cdc2/PP2A balance. Proc. Natl. Acad. Sci. USA. 107, 12564–12569 (2010). 20538976 10.1073/pnas.0914191107 PMC2906566

[b3] Gharbi-Ayachi A. . The substrate of Greatwall kinase, Arpp19, controls mitosis by inhibiting protein phosphatase 2A. Science 330, 1673–1677 (2010). 21164014 10.1126/science.1197048

[b4] Mochida S., Maslen S. L., Skehel M. & Hunt T. Greatwall phosphorylates an inhibitor of protein phosphatase 2A that is essential for mitosis. Science 330, 1670–1673 (2010). 21164013 10.1126/science.1195689

[b5] Álvarez-Fernández M. . Greatwall is essential to prevent mitotic collapse after nuclear envelope breakdown in mammals. Proc. Natl. Acad. Sci. USA. 110, 17374–17379 (2013). 24101512 10.1073/pnas.1310745110 PMC3808628

[b6] Wang P. . Cell cycle regulation of Greatwall kinase nuclear localization facilitates mitotic progression. J. Cell Biol. 202, 277–293 (2013). 23857770 10.1083/jcb.201211141 PMC3718974

[b7] Peters J.-M. The anaphase-promoting complex: proteolysis in mitosis and beyond. Mol. Cell 9, 931–943 (2002). 12049731 10.1016/s1097-2765(02)00540-3

[b8] Gavet O. & Pines J. Progressive activation of CyclinB1-Cdk1 coordinates entry to mitosis. Dev. Cell 18, 533–543 (2010). 20412769 10.1016/j.devcel.2010.02.013 PMC3325599

[b9] Lindqvist A., van Zon W., Karlsson Rosenthal C. & Wolthuis R. M. F. Cyclin B1-Cdk1 activation continues after centrosome separation to control mitotic progression. PLoS Biol. 5, e123 (2007). 17472438 10.1371/journal.pbio.0050123 PMC1858714

[b10] Potapova T. A., Sivakumar S., Flynn J. N., Li R. & Gorbsky G. J. Mitotic progression becomes irreversible in prometaphase and collapses when Wee1 and Cdc25 are inhibited. Mol. Biol. Cell 22, 1191–1206 (2011). 21325631 10.1091/mbc.E10-07-0599 PMC3078080

[b11] Chen Q., Zhang X., Jiang Q., Clarke P. R. & Zhang C. Cyclin B1 is localized to unattached kinetochores and contributes to efficient microtubule attachment and proper chromosome alignment during mitosis. Cell Res. 18, 268–280 (2008). 18195732 10.1038/cr.2008.11

[b12] Yuan J. . Stable gene silencing of cyclin B1 in tumor cells increases susceptibility to taxol and leads to growth arrest *in vivo*. Oncogene 25, 1753–1762 (2006). 16278675 10.1038/sj.onc.1209202

[b13] Th’ng J. P. . The FT210 cell line is a mouse G2 phase mutant with a temperature-sensitive CDC2 gene product. Cell 63, 313–324 (1990). 2208288 10.1016/0092-8674(90)90164-a

[b14] Itzhaki J., Gilbert C. & Porter A. Construction by gene targeting in human cells of a “conditional” CDC2 mutant that rereplicates its DNA. Nat. Genet. 15, 258–265 (1997). 9054937 10.1038/ng0397-258

[b15] Diril M. K. . Cyclin-dependent kinase 1 (Cdk1) is essential for cell division and suppression of DNA re-replication but not for liver regeneration. Proc. Natl. Acad. Sci. USA. 109, 3826–3831 (2012). 22355113 10.1073/pnas.1115201109 PMC3309725

[b16] Vassilev L. T. . Selective small-molecule inhibitor reveals critical mitotic functions of human CDK1. Proc. Natl. Acad. Sci. USA. 103, 10660–10665 (2006). 16818887 10.1073/pnas.0600447103 PMC1502288

[b17] McCloy R. A. . Partial inhibition of Cdk1 in G2 phase overrides the SAC and decouples mitotic events. Cell Cycle 13, 1400–1412 (2014). 24626186 10.4161/cc.28401 PMC4050138

[b18] Kaufmann S. H., Desnoyers S., Ottaviano Y., Davidson N. E. & Poirier G. G. Specific proteolytic cleavage of poly (ADP-ribose) polymerase: an early marker of chemotherapy-induced apoptosis. Cancer Res. 53, 3976–3985 (1993). 8358726

[b19] Akopyan K. . Assessing kinetics from fixed cells reveals activation of the mitotic entry network at the S/G2 transition. Mol. Cell 53, 843–853 (2014). 24582498 10.1016/j.molcel.2014.01.031

[b20] Dunsch A. K., Linnane E., Barr F. A. & Gruneberg U. The astrin-kinastrin/SKAP complex localizes to microtubule plus ends and facilitates chromosome alignment. J. Cell Biol. 192, 959–968 (2011). 21402792 10.1083/jcb.201008023 PMC3063133

[b21] Manning A. L. . CLASP1, astrin and Kif2b form a molecular switch that regulates kinetochore-microtubule dynamics to promote mitotic progression and fidelity. EMBO J. 29, 3531–3543 (2010). 20852589 10.1038/emboj.2010.230 PMC2964175

[b22] Schmidt J. C. . Aurora B kinase controls the targeting of the Astrin-SKAP complex to bioriented kinetochores. J. Cell Biol. 191, 269–280 (2010). 20937697 10.1083/jcb.201006129 PMC2958477

[b23] Santaguida S., Tighe A., D’Alise A. M., Taylor S. S. & Musacchio A. Dissecting the role of MPS1 in chromosome biorientation and the spindle checkpoint through the small molecule inhibitor reversine. J. Cell Biol. 190, 73–87 (2010). 20624901 10.1083/jcb.201001036 PMC2911657

[b24] Bellanger S., de Gramont A. & Sobczak-Thépot J. Cyclin B2 suppresses mitotic failure and DNA re-replication in human somatic cells knocked down for both cyclins B1 and B2. Oncogene 26, 7175–7184 (2007). 17533373 10.1038/sj.onc.1210539

[b25] D’Angiolella V., Mari C., Nocera D., Rametti L. & Grieco D. The spindle checkpoint requires cyclin-dependent kinase activity. Genes Dev. 17, 2520–2525 (2003). 14561775 10.1101/gad.267603 PMC218146

[b26] Berns K. . A large-scale RNAi screen in human cells identifies new components of the p53 pathway. Nature 428, 431–437 (2004). 15042092 10.1038/nature02371

[b27] Mollinari C. . PRC1 is a microtubule binding and bundling protein essential to maintain the mitotic spindle midzone. J. Cell Biol. 157, 1175–1186 (2002). 12082078 10.1083/jcb.200111052 PMC2173564

[b28] Neef R. . Choice of Plk1 docking partners during mitosis and cytokinesis is controlled by the activation state of Cdk1. Nat. Cell Biol. 9, 436–444 (2007). 17351640 10.1038/ncb1557

[b29] Kurasawa Y., Earnshaw W. C., Mochizuki Y., Dohmae N. & Todokoro K. Essential roles of KIF4 and its binding partner PRC1 in organized central spindle midzone formation. EMBO J. 23, 3237–3248 (2004). 15297875 10.1038/sj.emboj.7600347 PMC514520

[b30] Bieling P., Telley I. A. & Surrey T. A minimal midzone protein module controls formation and length of antiparallel microtubule overlaps. Cell 142, 420–432 (2010). 20691901 10.1016/j.cell.2010.06.033

[b31] Subramanian R. . Insights into antiparallel microtubule crosslinking by PRC1, a conserved nonmotor microtubule binding protein. Cell 142, 433–443 (2010). 20691902 10.1016/j.cell.2010.07.012 PMC2966277

[b32] Bringmann H. . A kinesin-like motor inhibits microtubule dynamic instability. Science 303, 1519–1522 (2004). 15001780 10.1126/science.1094838

[b33] Nunes Bastos R. . Aurora B suppresses microtubule dynamics and limits central spindle size by locally activating KIF4A. J. Cell Biol. 202, 605–621 (2013). 23940115 10.1083/jcb.201301094 PMC3747307

[b34] Zhu C. & Jiang W. Cell cycle-dependent translocation of PRC1 on the spindle by Kif4 is essential for midzone formation and cytokinesis. Proc. Natl. Acad. Sci. USA. 102, 343–348 (2005). 15625105 10.1073/pnas.0408438102 PMC544298

[b35] Hu C.-K., Coughlin M., Field C. M. & Mitchison T. J. KIF4 regulates midzone length during cytokinesis. Curr. Biol. 21, 815–824 (2011). 21565503 10.1016/j.cub.2011.04.019 PMC3100440

[b36] Zhu C., Lau E., Schwarzenbacher R., Bossy-Wetzel E. & Jiang W. Spatiotemporal control of spindle midzone formation by PRC1 in human cells. Proc. Natl. Acad. Sci. USA. 103, 6196–6201 (2006). 16603632 10.1073/pnas.0506926103 PMC1458854

[b37] Ma H. T., Tsang Y. H., Marxer M. & Poon R. Y. C. Cyclin A2-cyclin-dependent kinase 2 cooperates with the PLK1-SCFbeta-TrCP1-EMI1-anaphase-promoting complex/cyclosome axis to promote genome reduplication in the absence of mitosis. Mol. Cell. Biol. 29, 6500–6514 (2009). 19822658 10.1128/MCB.00669-09 PMC2786869

[b38] Villerbu N., Gaben A.-M., Redeuilh G. & Mester J. Cellular effects of purvalanol A: a specific inhibitor of cyclin-dependent kinase activities. Int. J. Cancer 97, 761–769 (2002). 11857351 10.1002/ijc.10125

[b39] Yuan J. . Cyclin B1 depletion inhibits proliferation and induces apoptosis in human tumor cells. Oncogene 23, 5843–5852 (2004). 15208674 10.1038/sj.onc.1207757

[b40] Bhatia A. & Kumar Y. Cancer cell micronucleus: an update on clinical and diagnostic applications. APMIS 121, 569–581 (2013). 23278233 10.1111/apm.12033

[b41] Luzhna L., Kathiria P. & Kovalchuk O. Micronuclei in genotoxicity assessment: from genetics to epigenetics and beyond. Front. Genet. 4, 1–17 (2013). 10.3389/fgene.2013.00131 PMC3708156 23874352

[b42] Decordier I., Cundari E. & Kirsch-Volders M. Survival of aneuploid, micronucleated and/or polyploid cells: crosstalk between ploidy control and apoptosis. Mutat. Res. 651, 30–39 (2008). 18242119 10.1016/j.mrgentox.2007.10.016

[b43] Gascoigne K. E. & Taylor S. S. Cancer cells display profound intra- and interline variation following prolonged exposure to antimitotic drugs. Cancer Cell 14, 111–122 (2008). 18656424 10.1016/j.ccr.2008.07.002

[b44] Jiang W. . PRC1: a human mitotic spindle-associated CDK substrate protein required for cytokinesis. Mol. Cell 2, 877–885 (1998). 9885575 10.1016/s1097-2765(00)80302-0

[b45] Cundell M. J. . The BEG (PP2A-B55/ENSA/Greatwall) pathway ensures cytokinesis follows chromosome separation. Mol. Cell 52, 393–405 (2013). 24120663 10.1016/j.molcel.2013.09.005 PMC3898901

[b46] Goga A., Yang D., Tward A. D., Morgan D. O. & Bishop J. M. Inhibition of CDK1 as a potential therapy for tumors over-expressing MYC. Nat. Med. 13, 820–827 (2007). 17589519 10.1038/nm1606

[b47] Brummelkamp T. R. . An shRNA barcode screen provides insight into cancer cell vulnerability to MDM2 inhibitors. Nat. Chem. Biol. 2, 202–206 (2006). 16474381 10.1038/nchembio774

[b48] Cong L. . Multiplex genome engineering using CRISPR/Cas systems. Science 339, 819–823 (2013). 23287718 10.1126/science.1231143 PMC3795411

[b49] Auer T. O., Duroure K., Cian A., De, Concordet J.-P. & Bene F. Del. Highly efficient CRISPR/Cas9-mediated knock-in in zebrafish by homology-independent DNA repair. Genome Res. 24, 142–153 (2014). 24179142 10.1101/gr.161638.113 PMC3875856

[b50] Bird A. W. & Hyman A. A. Building a spindle of the correct length in human cells requires the interaction between TPX2 and Aurora A. J. Cell Biol. 182, 289–300 (2008). 18663142 10.1083/jcb.200802005 PMC2483532

