# Supplementary material for: The lethal response to Cdk1 inhibition depends on sister chromatid alignment errors generated by KIF4 and isoform 1 of PRC1
Source: Sci Rep. 2015 Oct 1;5:14798. doi: 10.1038/srep14798 (PMC4589785; doi:10.1038/srep14798)
Supplement: Supplementary Information [file srep14798-s1.pdf]

The lethal response to Cdk1 inhibition depends on sister chromatid alignment errors generated by KIF4 and isoform 1 of PRC1

Authors: Erik Voets[1], Judith Marsman[1], Jeroen Demmers[2], Roderick Beijersbergen[1] and Rob Wolthuis[1, 3,*]

Affiliations:

[1] Division of Cell Biology I (B5) and Division of Molecular Carcinogenesis (B7), The Netherlands Cancer Insitute (NKI-AvL), Plesmanlaan 121, 1066 CX Amsterdam, The Netherlands;

[2] Proteomics Center, Erasmus University Medical Center, Dr Molewaterplein 50, 3015 GE Rotterdam, The Netherlands;

[3] Section of Oncogenetics, Department of Clinical Genetics and CCA/V-ICI Research Program Oncogenesis, VUmc Medical Faculty, van der Boechorststraat 7, 1081 BT Amsterdam, The Netherlands.

Supplementary information:

Supplementary Figures 1 to 6

Supplementary Tables 1 to 3

Voets et al. Supplementary Figure 1

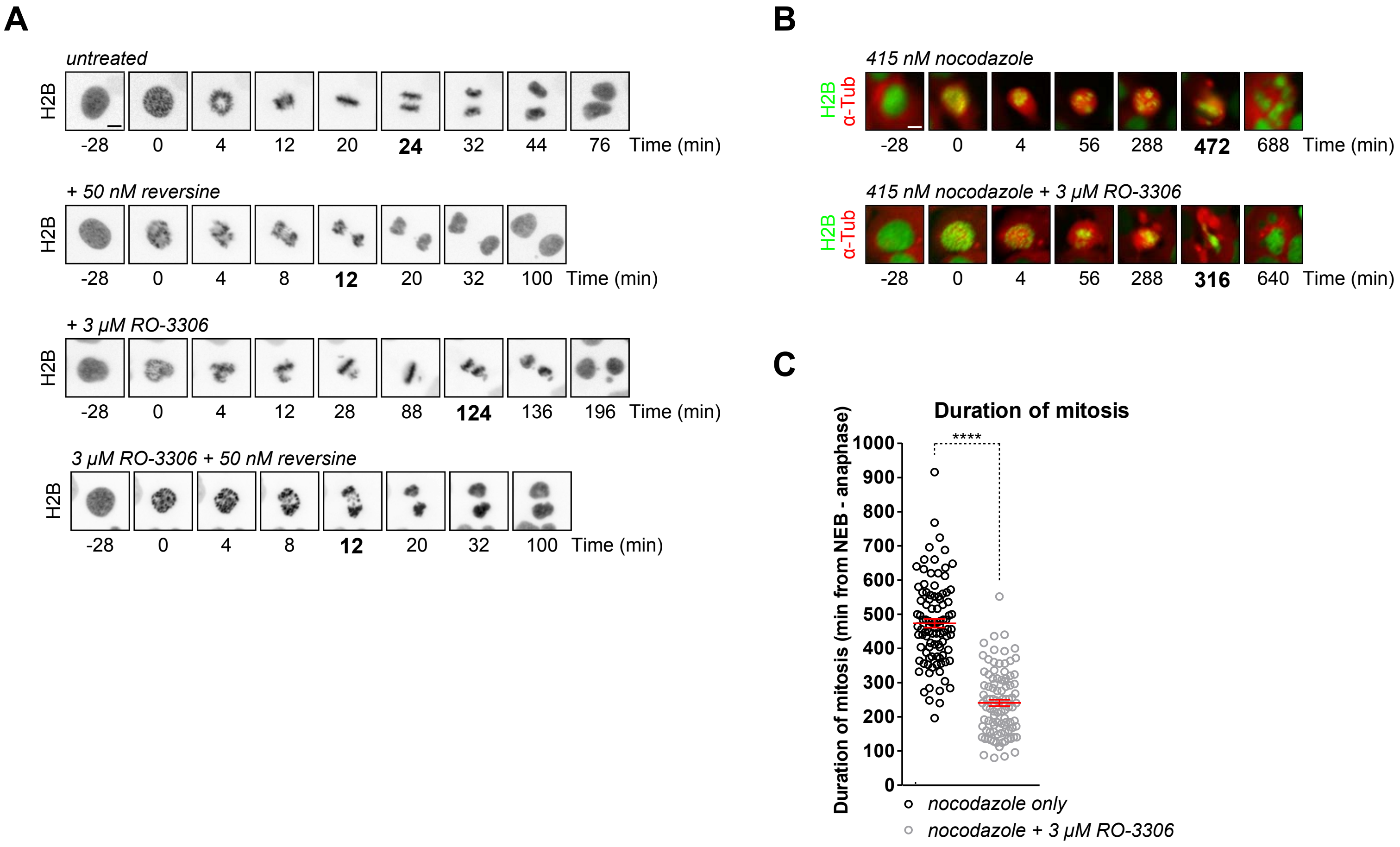

Voets et al. Supplementary Figure 2

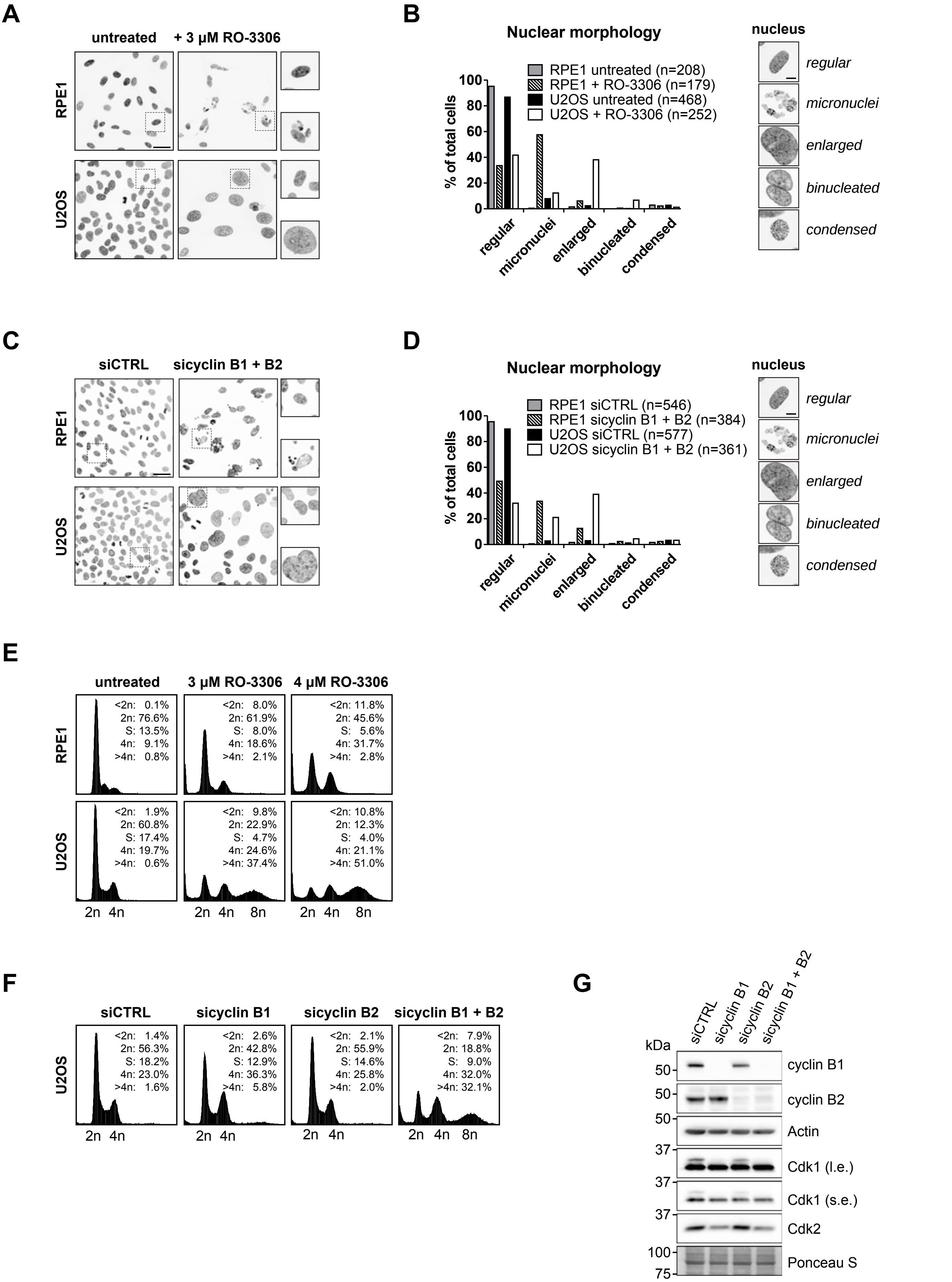

Voets et al. Supplementary Figure 3

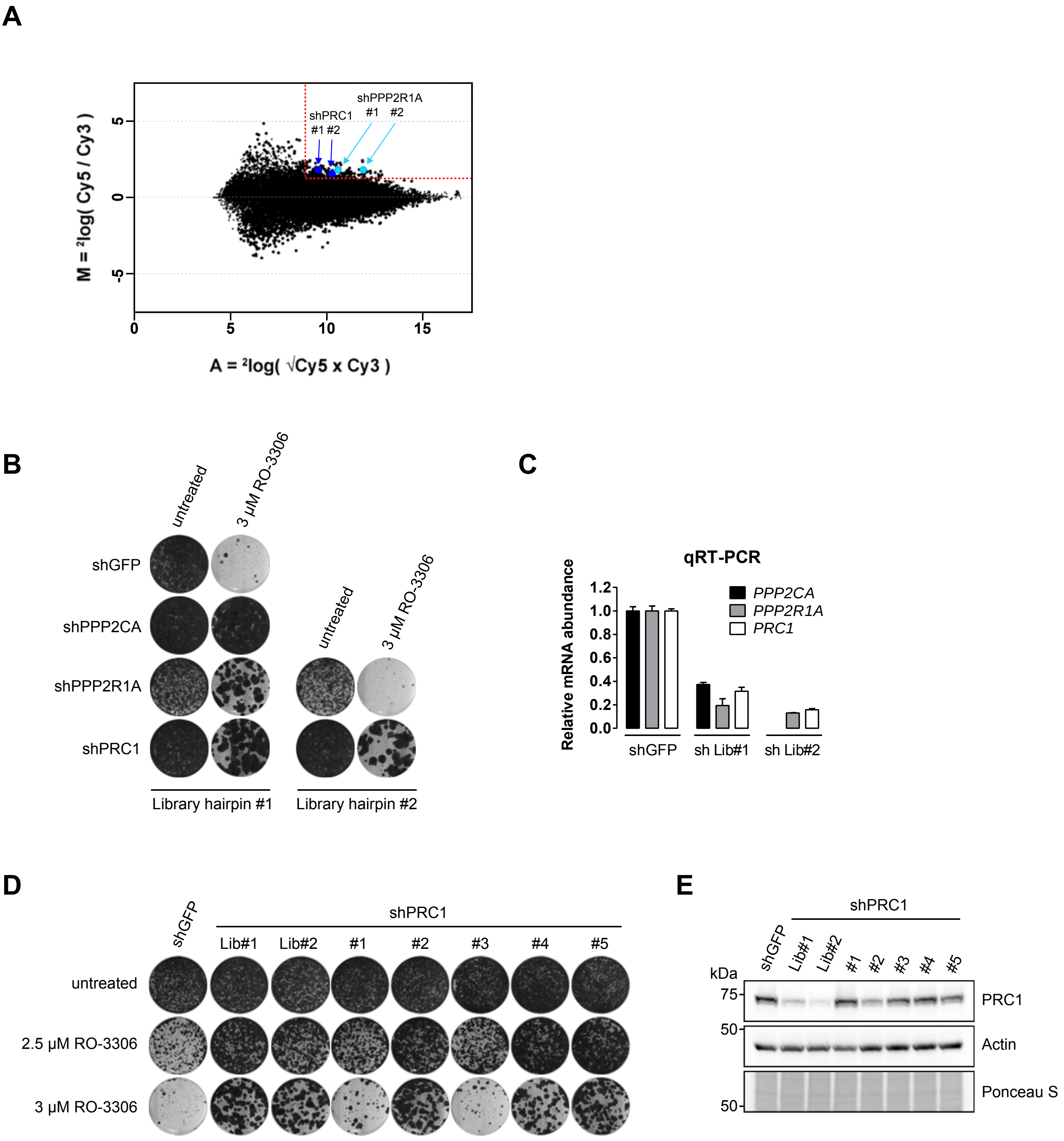

Voets et al. Supplementary Figure 4

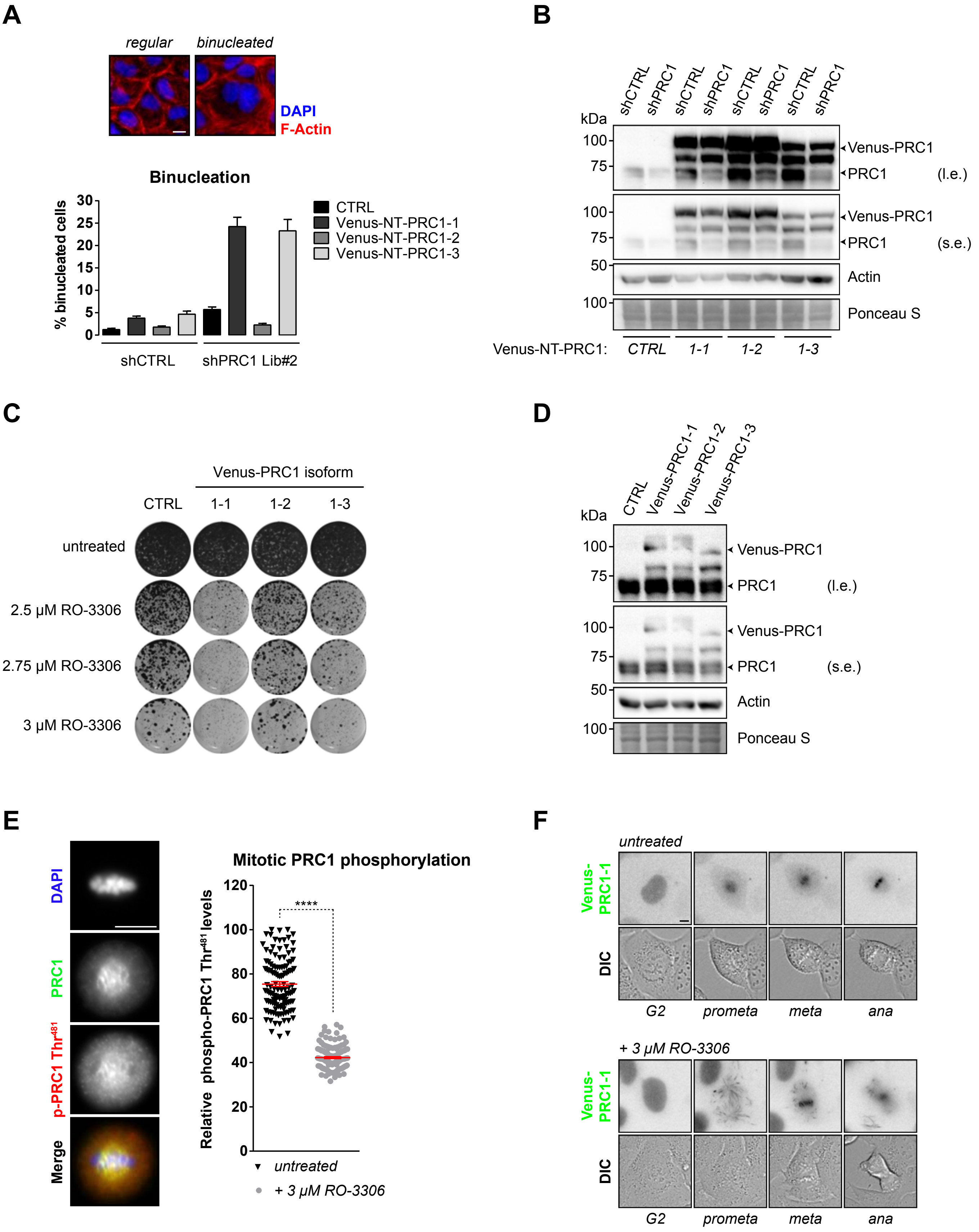

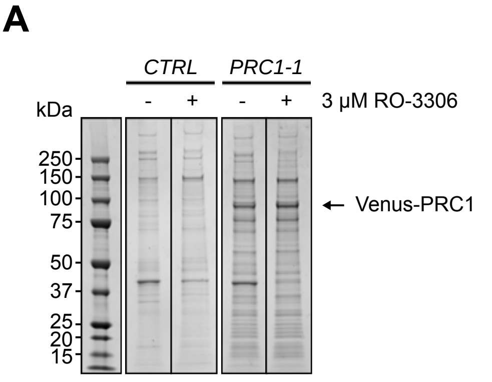

Protein identity	Venus-PRC1-1 untreated		Venus-PRC1-1 + RO-3306		
(MW in kDa)	Mascot	emPAI	Mascot	emPAI	Comments
PRC1 (71)	2614	26,25	2789	26.25	Protein regulator of cytokinesis 1
**KIF4A (140)**	4515	11.13	5250	16.85	Chromosome-associated kinesin KIF4A
**TUBB4A (50)**	1038	6.49	1033	5.49	Tubulin beta-4A chain
**CENPE (316)**	4838	2.23	6140	3.67	Centromere-associated protein E
**G3BP2 (54)**	772	3.05	409	1.38	Ras GTPase-activating protein-binding protein 2
**NAT10 (116)**	405	0.32	997	0.99	N-acetyltransferase 10
**LMNB1 (66)**	-	-	247	0.38	Lamin-B1
**NUMA1 (238)**	53	0.03	801	0.34	Nuclear mitotic apparatus protein 1
**USP10 (87)**	246	0.39	330	0.34	Ubiquitin carboxyl-terminal hydrolase 10
**CLASP1 (163)**	361	0.19	524	0.28	CLASP1 protein
**KIF23 (109)**	241	0.22	415	0.26	Kinesin-like protein KIF23
**CKAP5 (225)**	56	0.23	353	0.17	Cytoskeleton-associated protein 5
**CEP55 (54)**	159	0.3	79	0.14	Centrosomal protein of 55 kDa

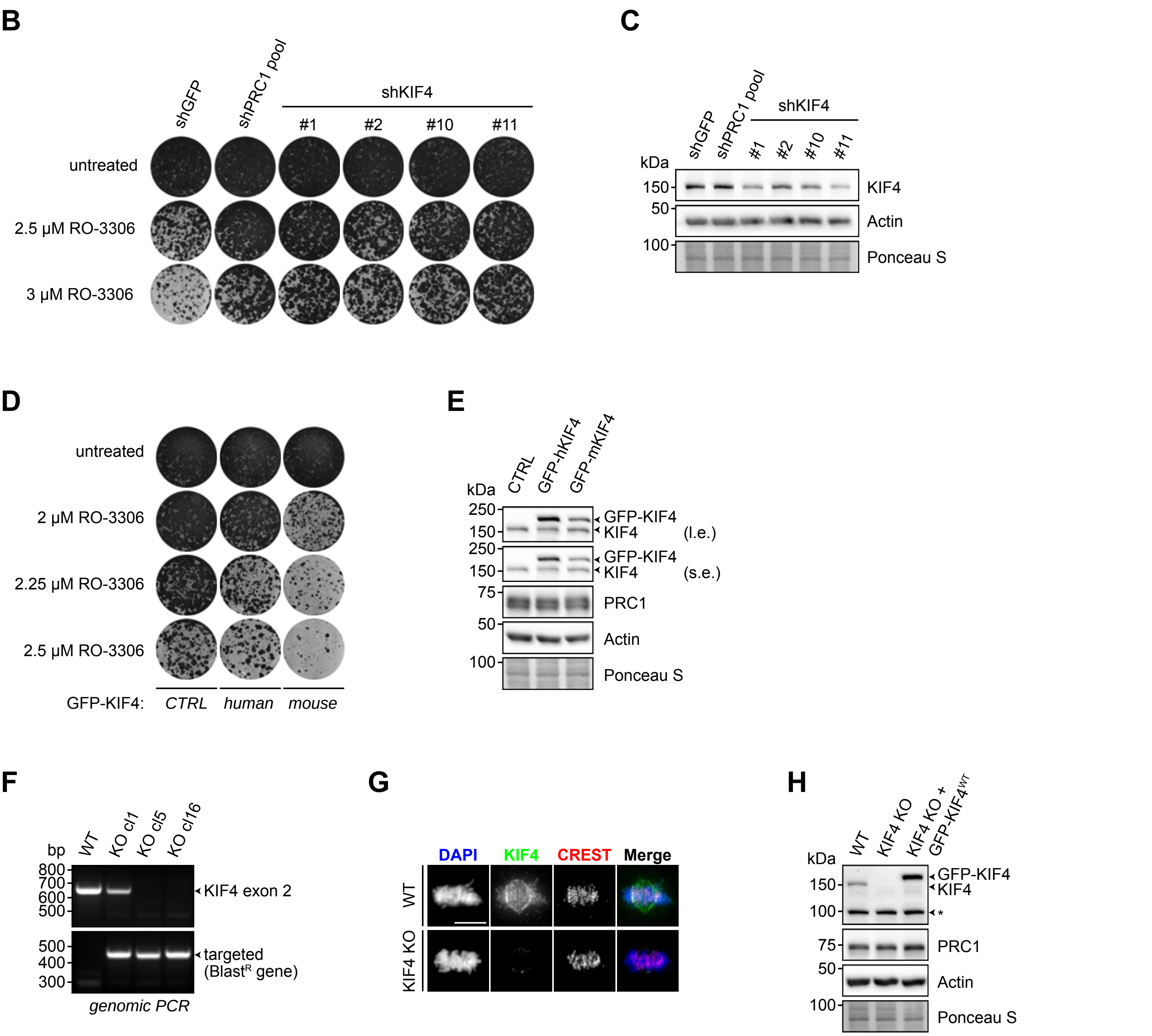

Voets et al. Supplementary Figure 6

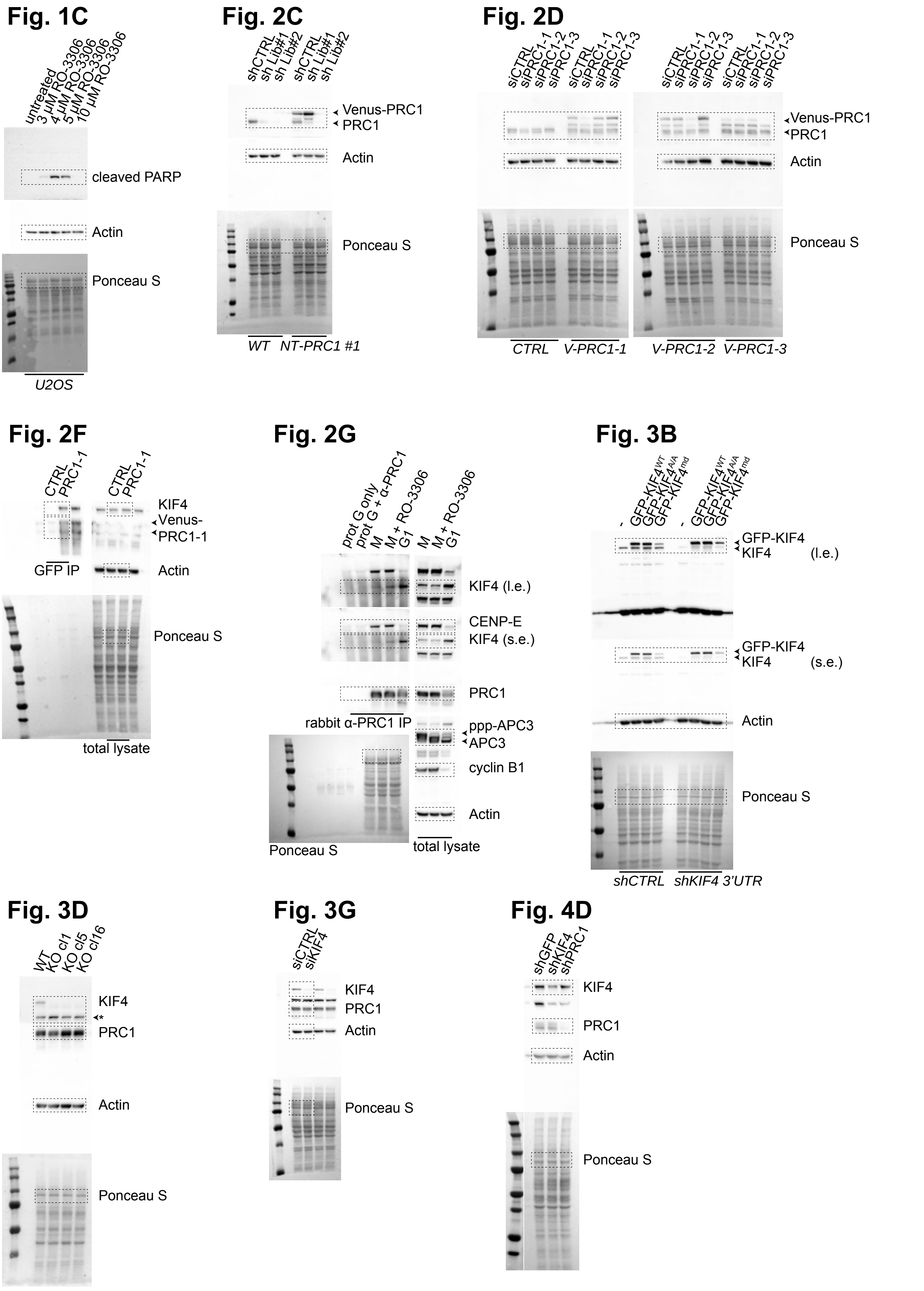

Supplementary Figure Legends

Supplementary Figure 1 | Cdk1 inhibition compromises the strength of the spindle checkpoint. (**A**) Mitosis in presence of a low dose of RO-3306 results in a spindle checkpoint-dependent mitotic delay. U2OS cells stably expressing H2B-GFP were followed by time-lapse imaging after treatment with indicated drugs. Scale bar, 10 µm. (**B**) Partial Cdk1 inhibition compromises the spindle checkpoint. U2OS cells stably expressing H2B-GFP and mCherry-α-Tubulin were followed in presence of 415 nM nocodazole and treated with or without 3 µM RO-3306. Scale bar, 10 µm. (**C**) Quantification of the mitotic duration after partial Cdk1 inhibition in presence of nocodazole. Mitosis is plotted from NEB till anaphase. (Mean ± s.e.m, *n* = 3, 32 cells/experiment; ****$p < 0.0001$, Student's *t* test).

Supplementary Figure 2 | Cyclin B RNAi resembles the cell cycle effects of the cyclin B-Cdk1 inhibitor RO-3306. (**A**) Comparison of the cell cycle effect of long-term RO-3306 treatment in RPE1 and U2OS cells, based on the nuclear morphology of the cells. Images show DAPI stainings. Scale bar, 50 µm. (**B**) Long-term Cdk1 inhibition results in endoreduplication in U2OS cancer cells, but not in non-transformed RPE1 cells. Quantification of the nuclear morphology of cells shown in (A). Examples of the different categories are shown in the panel on the right. n, amount of cells quantified per condition. Scale bar, 10 µm. (**C**) Comparison of the cell cycle effects of combined cyclin B1 and cyclin B2 RNAi in RPE1 and U2OS cells, based on the nuclear morphology of the cells. Images show DAPI stainings of a representative field of cells. Scale bar, 50 µm. (**D**) Suppression of cyclin B-Cdk1 activity by cyclin B depletion results in endoreduplication in U2OS cancer cells, but not in non-transformed RPE1 cells. Quantification of the nuclear morphology of cells shown in (C). Examples of the different categories are shown in the panel on the right. n, amount of cells quantified per condition. Scale bar, 10 µm. (**E**) Cell cycle profiles of RPE1 and U2OS cells treated with increasing doses of RO-3306. Cells were

analysed by flow cytometry 58 hours after RO-3306 treatment. Percentages of the different cell cycle phases are shown. The subG1 population (<2n) refers to non-viable cells. (**F**) Cell cycle profiles of U2OS cells after RNAi of cyclin B1, cyclin B2 or a combination of both. Percentages of the different cell cycle phases are shown. (**G**) Western blot analysis of protein lysates corresponding to cells transfected with siRNA duplexes targeting cyclin B1 and cyclin B2.

Supplementary Figure 3 | Suppression of PP2A and PRC1 rescues the long-term anti-proliferative effects of RO-3306. (**A**) Experimental output of the relative abundance of shRNAs recovered from the RO-3306 barcode screen. Averaged data from three independent experiments were normalized and ^{2}log transformed. Outliers from the barcode screen were selected for genes targeted by at least two independent shRNAs (M > 1 and A > 8.5). (**B**) Validation of the independent shRNAs targeting PPP2R1A and PRC1 in conferring resistance against RO-3306. The functional phenotypes of each individual shRNA are indicated by the colony formation assay in 3 μM RO-3306. The pRS vector targeting GFP (shGFP) was used as a control. (**C**) The knockdown efficiency of each individual shRNA was measured by examining the mRNA level of the corresponding target gene by qRT-PCR. Lib, library hairpin. (Mean ± s.d. *n* = 3). (**D**) Validation of multiple shRNAs targeting PRC1 in conferring resistance against RO-3306. The non-overlapping shRNAs #1 until #5 target the 3'UTR of the PRC1 mRNA. The pRS vector targeting GFP (shGFP) was used as a control. (**E**) Protein lysates corresponding to the colony formation assay in (D) analysed by Western blotting. The Western blot shows the knockdown efficiency of each of the non-overlapping shRNAs targeting PRC1.

Supplementary Figure 4 | Expression of PRC1 isoform 1 determines the sensitivity to RO-3306. (**A**) PRC1 splice variant 2 (PRC1-2) is the major cytokinesis regulator. WT (CTRL) or PRC1 knockdown (shPRC1 Lib#2) U2OS cells were fixed and stained after introduction of the shRNA-resistant Venus-PRC1 splice variants (Venus-NT-PRC1). Bar graph shows a

quantification of the percentage of binucleated cells. Insets demonstrate regular and binucleated cells. Scale bar, 10 µm. (Mean ± s.e.m, n = 3, at least 340 cells/experiment). (**B**) Protein lysates corresponding to the binucleation assay shown in (A) analysed by Western blotting. (**C**) Overexpression of mainly PRC1-1, but also PRC1-3, sensitizes cells towards Cdk1 inhibitor treatment. The functional phenotypes of overexpression of each of the individual Venus-tagged PRC1 splice variants are indicated by the colony formation assay. CTRL refers to cells that do not express Venus-PRC1. (**D**) Protein lysates corresponding to the colony formation assay shown in (C). (**E**) Mitotic PRC1 phosphorylation is diminished upon RO-3306 treatment. U2OS cells, treated with or without 3 µM RO-3306 for 16 hours, were fixed in ice-cold methanol to monitor mitotic PRC1 phosphorylation on Thr^{481}. ****$p < 0.0001$, Student's t test. Scale bar, 10 µm. (**F**) Partial Cdk1 inhibition drastically changes the localization of PRC1-1 in mitosis. U2OS cells stably expressing Venus-PRC1-1 were imaged by DIC and fluorescence microscopy. Cells were followed in presence or absence of 3 µM RO-3306, added at the onset of the experiment. Scale bar, 10 µm.

Supplementary Figure 5 | KIF4 is a mediator of cellular toxicity, induced by Cdk1 inhibition. (**A**) Mass spectrometry to identify binding partners of PRC1-1. IPs were performed from extracts of U2OS WT or U2OS cells stably expressing Venus-PRC1-1 untreated or treated with 3 µM RO-3306 for 64 hours using GFP-Trap_A beads. Aliquots of the IPs were analysed by SDS-PAGE and stained with Colloidal blue. Ranking of potential PRC1-1 interactors is based on the emPAI score. (**B**) KIF4 knockdown confers resistance against RO-3306. The functional phenotypes of non-overlapping shRNAs targeting KIF4 are indicated by the colony formation assay. shPRC1 pool refers to a combined set of four individual shRNAs targeting PRC1. The pRS vector targeting GFP (shGFP) was used as a control. (**C**) Protein lysates corresponding to the colony formation assay in (B) analysed by Western blotting. (**D**) Overexpression of KIF4 specifically sensitizes cells towards Cdk1 inhibitor treatment. The functional phenotypes of

U2OS cells overexpressing human or mouse GFP-KIF4 are indicated by the colony formation assay. CTRL refers to cells that do not express GFP-KIF4. (**E**) Protein lysates corresponding to the colony formation assay in (D) analysed by Western blotting. (**F**) U2OS WT and KIF4 KO clones were characterized by PCR demonstrating correct targeting and the resulting loss of KIF4 protein. (**G**) U2OS WT and KIF4 KO clone 16 were immunostained for KIF4 (green), kinetochores (red), and DNA (blue). Scale bar, 10 µm. (**H**) Verification of the GFP-KIF4^{WT} reconstitution in KIF4 KO cells judged by Western blot analysis.

Supplementary Figure 6 | Uncropped Western blot images. Western blots with corresponding main figure numbers are shown. Dashed boxes highlight the cropped segment presented in the main figures.

Supplementary Table 1 | shRNA sequences used in this study

gene name	accession_number	hairpin	targeting sequence (5’ → 3’)
GFP	P42212		GCTGACCCTGAAGTTCATC
KIF4	NM_012310	#1	AAGCAGATTGAAAGCCTAGAG
KIF4	NM_012310	#2	GCTTCAAGATTCTCTAGGA
KIF4	NM_012310	#3	CAATTGATTACCCAGTTAT
KIF4	NM_012310	#9	AGGCGTACATTCTCCCTTA
KIF4	NM_012310	#10	GAAGAGGCCCACTGAAGTT
KIF4	NM_012310	#11	TGAAAGAGATGTGCGATGT
KIF4	NM_012310	#12	TGACTCGACTGCTTCAAGA
KIF4	NM_012310	3'UTR	CAGGTCCAGACTACTACTC
PPP2CA	NM_002715	Lib#1	GGATAGCAGCAAACAATCA
PPP2R1A	NM_014225	Lib#1	GAAGCTGTCCACCATCGCC
PPP2R1A	NM_014225	Lib#2	GGCGGAACTTCGACAGTAC
PRC1	NM_199414	Lib#1	GAAGGAGAGACGACCATCT
PRC1	NM_199414	Lib#2	GTGATTGAGGCAATTCGAG
PRC1	NM_199414	#1	CATGAAAAGTTTATGGCAA
PRC1	NM_199414	#2	GTTTAGGCATGTTCAATTA
PRC1	NM_199414	#3	GGGTATGCATGTATGAACA
PRC1	NM_199414	#4	GGGCCTAACTTAAAGATGT
PRC1	NM_199414	#5	GTTTAGTGGTCCTAGGAGA
PRC1-1	NM_003981	#1-1	GAGTTTGCGAAGGATCCGT
PRC1-2	NM_199413	#1-2	GTTTGCGCGAGAACTTTCA
PRC1-3	NM_199414	#1-3	ATCCTGAGTGCGAGAACTT

Supplementary Table 2 | siRNA sequences used in this study

gene name	aliases	accession_number	oligo	targeting sequence
CCNB1	cyclin B1	NM_031966	J-003206-09	CAACAUUACCUGUCAUAUA
CCNB1	cyclin B1	NM_031966	J-003206-10	UGCACUAGUUCAAGAUUUA
CCNB1	cyclin B1	NM_031966	J-003206-11	GAAUGUAGUCAUGGUAAAU
CCNB1	cyclin B1	NM_031966	J-003206-12	CUAAUUGACUGGCUAGUAC
CCNB2	cyclin B2	NM_004701	J-003207-09	GUGACUACGUUAAGGAUAU
CCNB2	cyclin B2	NM_004701	J-003207-10	GUACAUGUGCGUUGGCAUU
CCNB2	cyclin B2	NM_004701	J-003207-11	CAAGUCCACUCCAAGUUUA
CCNB2	cyclin B2	NM_004701	J-003207-12	UAACGAAGAUUGGGAGAAC
KIF4A	KIF4	NM_012310	J-004961-09	AGGCGUACAUUCUCCCUUA
KIF4A	KIF4	NM_012310	J-004961-10	GAAGAGGCCCACUGAAGUU
KIF4A	KIF4	NM_012310	J-004961-11	UGAAAGAGAUGUGCGAUGU
KIF4A	KIF4	NM_012310	J-004961-12	UGACUCGACUGCUUCAAGA
PRC1	PRC1	NM_199414	J-019491-05	ACAAAGAACUGAGGUGGUA
PRC1	PRC1	NM_199414	J-019491-06	GCACGUAAGCUGAACACUA
PRC1	PRC1	NM_199414	J-019491-07	CCGGAAAGCGCUGCAAUUA
PRC1	PRC1	NM_199414	J-019491-08	UAAAUCACCUUCGGGAAAU
PRC1-1	variant 1	NM_003981	custom	GAGUUUGCGAAGGAUCCGU
PRC1-2	variant 2	NM_199413	custom	GUUUGCGCGAGAACUUUCA
PRC1-3	variant 3	NM_199414	custom	AUCCUGAGUGCGAGAACUU

Supplementary Table 3 | qRT-PCR primers used in this study

gene name	accession_number	forward primer (5' → 3')	reversed primer (5' → 3')
GAPDH	NM_002046	AAGGTGAAGGTCGGAGTCAA	AATGAAGGGGTCATTGATGG
PPP2CA	NM_002715	ACCTCTTGCACGTTGGATTC	AGTGGATCGAGCAGCTGAA
PPP2R1A	NM_014225	TCACTTCGGGTCCTTTCAAC	CTCATAGACGAACTCCGCAA
PRC1	NM_199414	TACTATGGACTCCTCCGCCA	CCGTGTGGAGTAGGTCTGGA
PRC1-1	NM_003981	CCCTCCAGCGCAACTTCAGCA	TCGCTGAAGCCCAACAGTGG
PRC1-2	NM_199413	CGGCAGCATCCTGAGTGGTGG	AGCCTTTGAAAGTTCTCGCGCA
